# An Interstitial 4q Deletion with a Mosaic Complementary Ring Chromosome in a Child with Dysmorphism, Linear Skin Pigmentation, and Hepatomegaly

**DOI:** 10.1155/2017/4894515

**Published:** 2017-07-27

**Authors:** J. Carter, H. Brittain, D. Morrogh, N. Lench, J. J. Waters

**Affiliations:** ^1^NE Thames Regional Genetics Service, Great Ormond Street Hospital for Children NHS Foundation Trust, London, UK; ^2^Congenica Ltd., Wellcome Trust Campus, Hinxton, Cambridge CB10 15A, UK

## Abstract

Interstitial deletions of 4q are rarely reported, vary in size, and have limited genotype-phenotype correlations. Here, genome-wide array CGH analysis identified a 21.6 Mb region of copy number loss at 4q12-q21.1 in a patient diagnosed with dysmorphism, linear skin pigmentation, and hepatomegaly. An additional small ring chromosome was detected in 5/30 cells examined via G-banding. Confirmation of the origin of the ring chromosome was obtained by FISH analysis which identified that the ring chromosome contained material from the deleted region of chromosome 4 and was therefore complementary to the 21.6 Mb deletion. Further microarray studies in the proband using a different microarray platform showed no evidence of mosaicism. This case highlights the importance of an integrated approach to cytogenetic analysis and demonstrates the value of G-banding for detecting mosaicism, as current microarray platforms are unable to detect low level mosaics.

## 1. Introduction

Microarray analysis is the first-line test for dysmorphism and/or developmental delay in the genetics laboratory, but other techniques can be useful in addition to this method depending upon the clinical features of the patient and the type of abnormality detected. Interstitial deletions of 4q have been reported previously with variability in size, gene content, and phenotype; however deletions with a complimentary ring chromosome have not been reported previously in the literature in a postnatal context.

## 2. Case Presentation

The patient was referred for microarray analysis at six months of age. He presented with upslanting palpebral fissures, upturned nose and earlobes, divergent squint, talipes, hepatomegaly linear skin pigmentation, and developmental delay. His cardiac assessment was normal, and height and weight are on the 2nd–9th centile.

**Figure 1:**
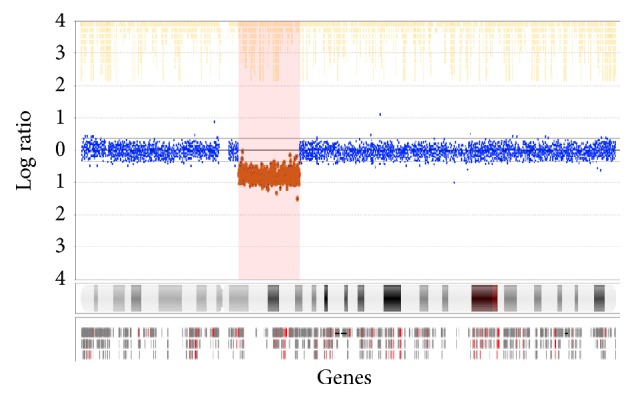
Nimblegen 135 K WG CGH v.3.1 microarray, chromosome 4.

Genome-wide aCGH analysis on DNA extracted from peripheral blood was carried out using the Nimblegen 135 K WG CGH v.3.1 platform (Figure 1). This identified a 21.6 Mb deletion:

  arr[HG19] 4q12q21.1(56288188_77876928)x1.

**Figure 2:**
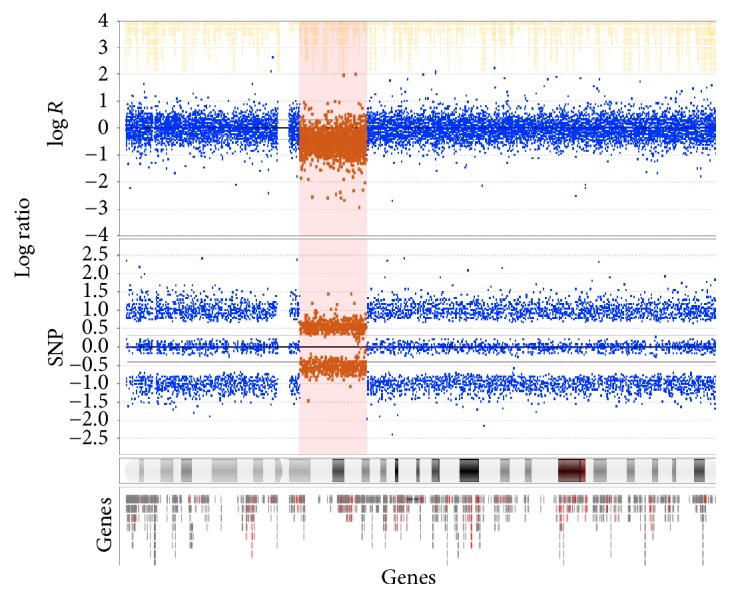
Affymetrix 750 K SNP Array, chromosome 4.

This finding was confirmed using a separate microarray platform, the Affymetrix 750 K SNP Array (Figure 2).

Due to the patient's linear skin pigmentation, an initial 30-cell G-banding screen from lymphocyte culture was carried out alongside the microarray analysis to rule out the possibility of mosaicism. 25/30 cells examined contained an interstitial deletion of the long arm of chromosome 4, and 5/30 cells (16.7%) were found to contain a ring chromosome in addition to the deleted chromosome 4. There was no evidence of mosaicism from either microarray platform.

**Figure 3:**
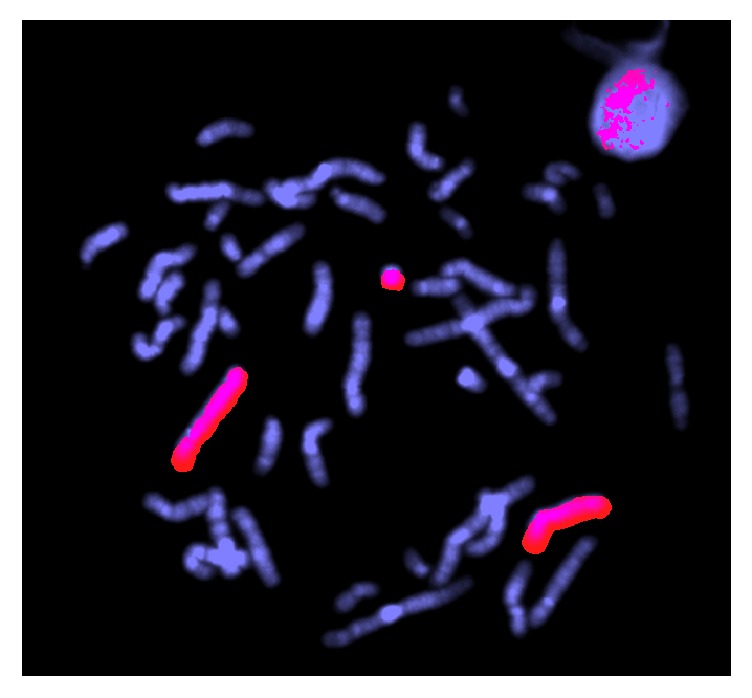
WCP probe for chromosome 4.

FISH analysis was instigated to confirm the origin of the ring chromosome. Whole chromosome paints demonstrated that the ring originated from chromosome 4 (Figure 3).

**Figure 4:**
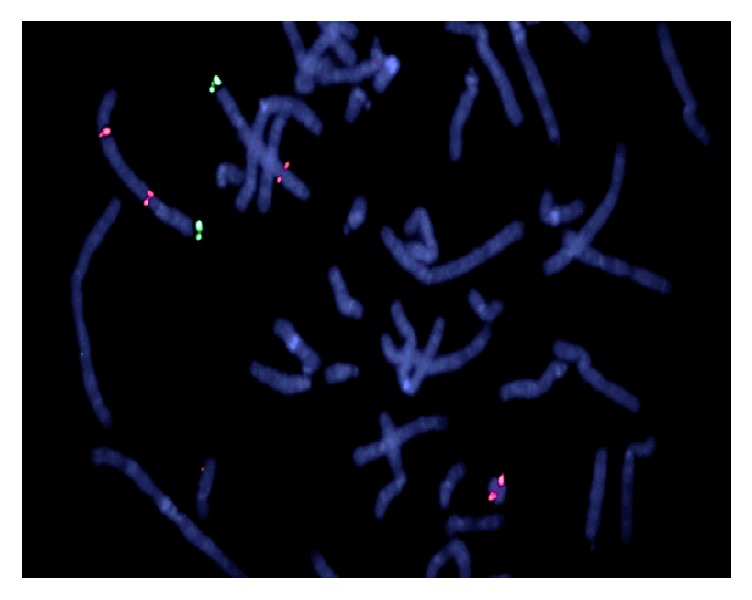
Red signal = RP11-158016 4q13.1 (also hybridizes to 4q31); green signal = 4 pter.

The BlueGnome FISH probe RP11-158016 for the region 4q13.1 confirmed the deletion and the presence of 4q material from this region on the ring chromosome (Figure 4).

Parental karyotyping and FISH identified the rearrangement as de novo in origin.

The combination of these three cytogenetic techniques produced the following final ISCN:

  46,XY,del(4)(q12q21.1).arr[HG19]4q12q21.1(56288188_77876928)x1dn[25]/47,XY,del(4)(q12q21.1),+r(4)(q12q21.1)dn[5].

At 22 months, the patient was pulling to stand and had a few words.

## 3. Discussion

The region of deletion contains 129 genes, none of which have any clear association with the patient's phenotype in the literature. One gene, EPHA5, has been linked to neuronal development, in particular synapse formation in mouse models [1]. This gene has a haploinsufficiency (HI) score of 5.72% which indicates that this gene is predicted to have a dosage effect. UBA6 (ubiquitin-like modifier-activating enzyme 6) has been associated with a role in neurological developmental and behaviour disorders in mice models, has a HI score of 26.21%, and therefore may have a dosage effect [2].

Only one similar case of a mosaic complementary ring chromosome 4 has been previously reported [3]. The region of deletion was approximately 22 Mb at 4q11q13.3 with a complementary ring chromosome identified by G-banding and FISH in 9 out of 13 cells. This was a prenatal finding with no abnormalities on ultrasound scan and no information on the pregnancy outcome.

Patients with similar-sized deletions of this region (but without a complementary ring chromosome) have been reported with moderate to severe mental retardation, minor facial anomalies, and growth retardation [4]. Some patients also have a piebald trait which has been mapped to the KIT gene [5] at 4q12; however Hemati et al. [6] reported a patient with KIT haploinsufficiency and absence of piebaldism which suggests that the role of KIT is not necessarily a simple dosage effect. This gene was not deleted in our patient; therefore it is likely that the linear skin pigmentation which prompted the mosaicism screen was due to the patient's mosaic status rather than the genetic content of the deletion. No similar deletions have been reported previously in our laboratory.

Smaller deletions have also been recorded in association with mild intellectual disability, and UBA6 was also proposed as a candidate gene [7].

**Figure 5:**
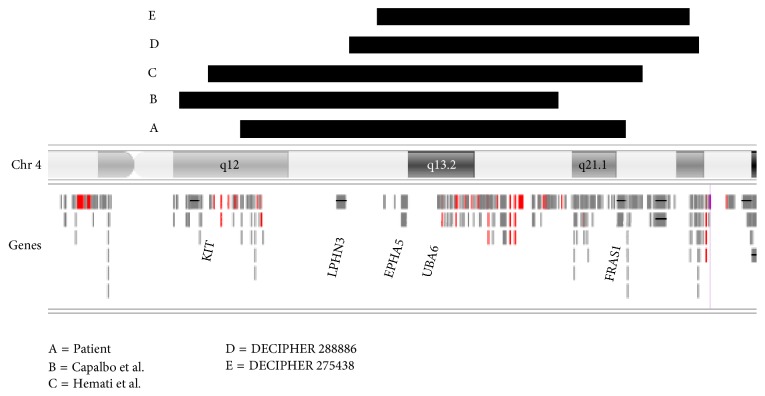
Comparison of 4q11-q21.1 deletion patients from the literature and databases.

The DECIPHER database revealed a patient (ref. 288886) with a similar deletion (21.48 Mb) and intellectual disability recorded as “likely pathogenic” and a patient with multiple congenital abnormalities and a smaller, 19 Mb, deletion (ref. 275438) with unknown inheritance and pathogenicity. See Figure 5 for a comparison of reported patients.

A search of the DGV (Database of Genomic Variants) indicated that copy number loss for this region has not been found in normal individuals.

This large deletion of 4q is likely to be contributing to the patient's phenotype, although a dosage effect of any of the genes involved has not yet been demonstrated. In general, large deletions of 4q have a less severe phenotype than might be expected given the large amount of genes involved which would be consistent with this patient's relatively mild features. The complementary ring chromosome may compensate for the deletion possibly resulting in a milder phenotype depending on the level of mosaicism across different tissues. It is unclear then if the phenotype is a result of compensatory mosaicism or purely the gene content of the deletion. As this is the second complementary ring reported for this region, it suggests that this region could be prone to recombination and other patients with complementary ring chromosomes could be underreported.

This case highlights the importance of an integrated approach to cytogenetic analysis, and as microarray analysis did not detect low level mosaicism (<20%) for the r(4) in this case, we recommend that if a patient phenotype is indicative of mosaicism both G-banded scoring and microarray analysis should be performed in parallel.
